# Global Epidemiology of Outbreaks of Unknown Cause Identified by Open-Source Intelligence, 2020–2022

**DOI:** 10.3201/eid3102.240533

**Published:** 2025-02

**Authors:** Damian Honeyman, Deepti Gurdasani, Adriana Notaras, Zubair Akhtar, Jared Edgeworth, Aye Moa, Abrar Ahmad Chughtai, Ashley Quigley, Samsung Lim, Chandini Raina MacIntyre

**Affiliations:** The University of New South Wales, Sydney, New South Wales, Australia (D. Honeyman, D. Gurdasani, A. Notaras, Z. Akhtar, J. Edgeworth, A. Moa, A.A. Chughtai, A. Quigley, S. Lim, C.R. MacIntyre); University of Western Australia, Perth, Western Australia, Australia (D. Gurdasani); Queen Mary University of London, London, UK (D. Gurdasani); Arizona State University, Tempe, Arizona, USA (C.R. MacIntyre)

**Keywords:** Artificial intelligence, open-source intelligence, epidemiology, disease, surveillance, outbreaks of unknown cause, worldwide, zoonoses, high-consequence pathogens

## Abstract

Epidemic surveillance using traditional approaches is dependent on case ascertainment and is delayed. Open-source intelligence (OSINT)–based syndromic surveillance can overcome limitations of delayed surveillance and poor case ascertainment, providing early warnings to guide outbreak response. It can identify outbreaks of unknown cause for which no other global surveillance exists. Using the artificial intelligence–based OSINT early warning system EPIWATCH, we describe the global epidemiology of 310 outbreaks of unknown cause that occurred December 31, 2019–January 1, 2023. The outbreaks were associated with 75,968 reported human cases and 4,235 deaths. We identified where OSINT signaled outbreaks earlier than official sources and before diagnoses were made. We identified possible signals of known disease outbreaks with poor case ascertainment. A cause was subsequently reported for only 14% of outbreaks analyzed; the percentage was substantially lower in lower/upper-middle–income economies than high-income economies, highlighting the utility of OSINT-based syndromic surveillance for early warnings, particularly in resource-poor settings.

Emerging and reemerging pathogens causing infectious diseases in human and animal populations are an ongoing and substantial public health threat. The threat is particularly relevant as climate change and land use alter patterns of illness and increase the frequency of high-consequence zoonotic infection spillover into human populations (*1*). Traditional surveillance systems (*2*) often rely on validated data from laboratories or hospitals. Approaches that do not depend on laboratory confirmation, such as clinical syndromic or sentinel surveillance, can provide earlier warnings of outbreaks and detect signals of newly emerged infections for which no diagnostics are yet available. Such approaches can overcome the lack of robust traditional surveillance and limited testing capabilities for many diseases in resource-poor settings.

Open-source epidemic intelligence (OSINT) is the collection, analysis, and use of information from open sources such as news media, websites, or social media (*3*). OSINT provides an alternative method for syndromic surveillance. Such surveillance can enhance early detection of novel emerging infections (e.g., SARS-CoV-2 in 2020), missed outbreaks of known pathogens, or delayed reporting via traditional surveillance.

Novel infections will initially emerge as an illness or syndrome of unknown cause before diagnostic tests are developed. If diagnostic capabilities are limited, even for known illnesses, a cause may not be identified for many outbreaks. According to a study of 109 outbreaks of unknown cause identified during 2016–2019, a cause was later confirmed for only 18% of the outbreaks; the most frequently identified pathogens were measles virus, Nipah virus, norovirus, and influenza virus (*4*). The lack of pathogen identification for >80% of outbreaks is concerning, underlining the value of syndromic surveillance. The outbreaks could be sporadic zoonotic outbreaks with no available diagnostics, emerging infections of pandemic potential, or outbreaks in low-resource countries with weak health systems.

Knowledge of the incidence, burden, and identified causes of outbreaks of unknown etiology is essential. Such data cannot be collected routinely because they are not collected by traditional surveillance methods. Syndromic surveillance could address that gap; however, when conducted as part of routine national surveillance programs, syndromic surveillance is often restricted to clinical rather than community settings and to specific syndromes only (e.g., influenza-like illness) (*5*). OSINT-based syndromic surveillance can provide broader insights into the frequency and outcomes of outbreaks of unknown cause across the globe, providing a useful epidemiologic tool for the timely detection of infectious disease outbreaks, thus providing rapid outbreak alerts, guiding early responses, and minimizing disease spread (*6*,*7*).

EPIWATCH (https://www.epiwatch.org) is an artificial intelligence–based surveillance system that collects and processes vast amounts of multilingual OSINT data from news media and publicly available online sources worldwide to provide early warnings about potential outbreaks (*8*). EPIWATCH has been evaluated extensively and found to be a valid tool for epidemic surveillance and early warnings (*8*–*10*). EPIWATCH provides accurate information regarding trends and case numbers, particularly when case ascertainment has been poor (*11*), making it ideal for broad syndromic surveillance.

We used the EPIWATCH database to analyze reports of unknown and syndromic disease outbreaks to describe the epidemiology of those reported outbreaks globally during 2020–2022. No ethics application was required for this study because publicly available aggregate data were used.

## Methods

### Search Strategy

**Figure 1:**
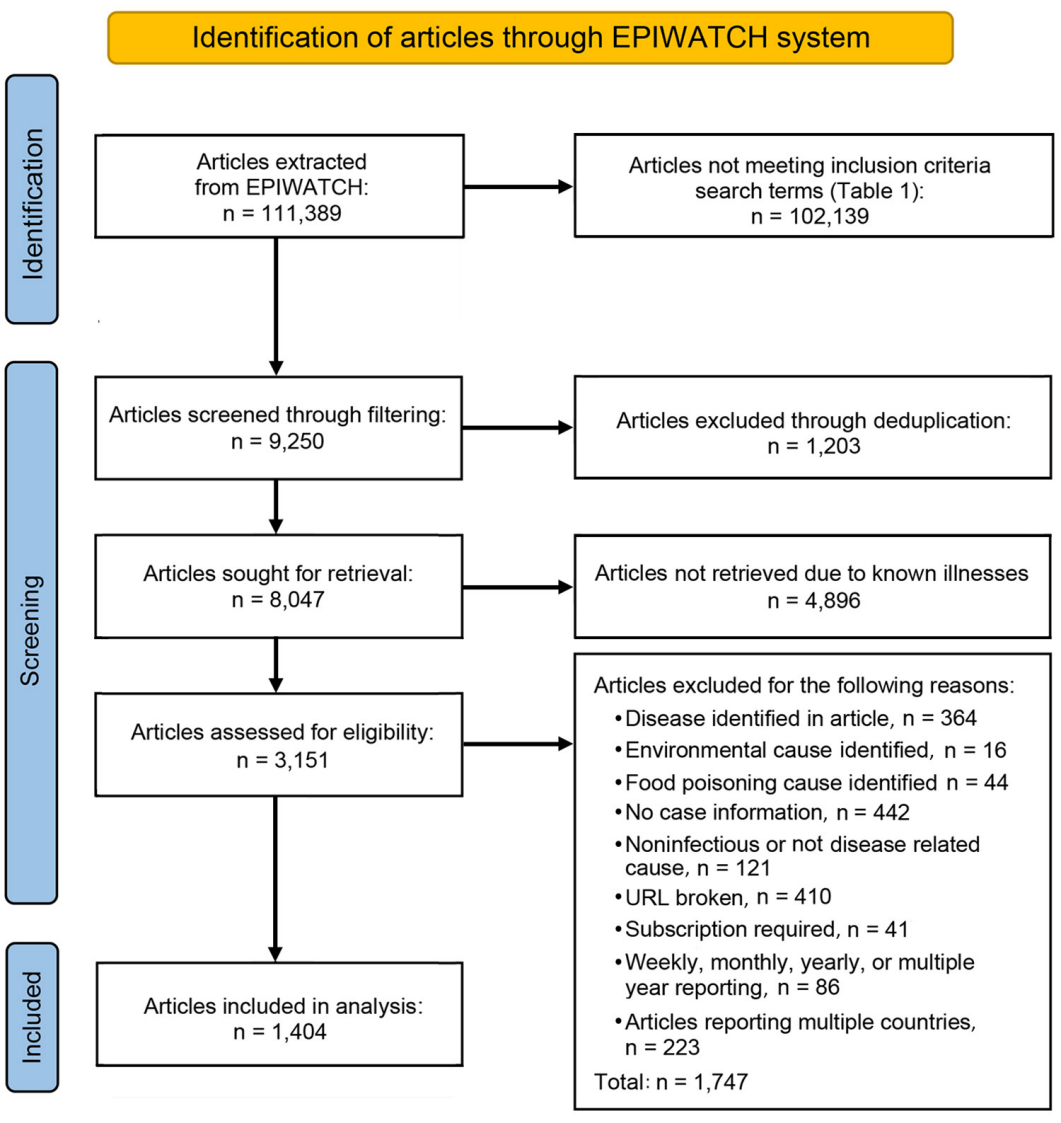
Articles extracted from EPIWATCH system (https://www.epiwatch.org), December 31, 2019–January 1, 2023.

We curated a list of search terms and syndromes reflecting outbreaks of unknown cause based on similar work from existing literature (*4*) and discussion within our expert panel (authors D.H., A.Q., A.C., and D.G.) to add relevant terms (Table 1; Figure 1). We retrieved a dataset from the EPIWATCH database by using those search terms to locate articles published during December 31, 2019–January 1, 2023. We defined an outbreak of unknown cause as an outbreak for which the cause was unknown at the time of the outbreak and the suspected cause was an infection with >2 linked cases.

**Table 1:** Search terms used for data retrieval and syndromes identified from EPIWATCH, 2020–2022

Category
Search terms
Acute flaccid paralysis
Acute gastroenteritis
Bronchiolitis
Bronchitis
Death
Die off
Encephalitis
Encephalomyelitis
Fever
Food poisoning
Illness
Influenza-like illness
Meningitis
Mortality
Mysterious
Mystery
Not known
Not specified
Not yet classified
Pneumonia
Rash
Suspicious
Severe acute respiratory illness
Suspicious
Unclassified
Unclear
Undiagnosed
Unexplained
Unidentified
Unknown

Syndromes
Acute flaccid paralysis
Acute gastroenteritis
Encephalitic
Febrile
Fever with rash
Respiratory*
Meningitis
Unknown†

*Includes influenza-like-illness, severe acute respiratory infection, and pneumonia. †Insufficient information in the article for classification.

### Inclusion and Exclusion Criteria

We filtered articles according to the presence of any of the prespecified syndrome-related search terms within the title (Table 1; Figure 1). We excluded articles about routine monthly, quarterly, or yearly syndromic surveillance (e.g., national pneumonia surveillance) because we wanted to capture articles via OSINT rather than official surveillance reports. We also excluded articles reporting on multicountry outbreaks because detections were likely to be later (after spread had occurred) and unlikely to constitute early warnings.

### Data Extraction

Four analysts (authors D.H., A.N., Z.A., and J.E.) extracted prespecified data from all eligible full-text articles. We first removed duplicated articles and then removed any inaccessible articles (e.g., broken URLs, subscription required), articles about outbreaks with a confirmed cause of illness, or articles that did not mention case numbers or location (Table 2; Figure 1). We extracted data for country, event date, state/province/city (where available), adult symptoms, child symptoms, syndrome, human or animal outbreak, case numbers, sex distribution of patients, and deaths. We classified syndromes into prespecified categories on the basis of the dominant clinical manifestations (Table 1). To ensure consistency and accuracy, we randomly checked 100 articles.

**Table 2:** Inclusion and exclusion criteria used in study of global epidemiology of outbreaks of unknown cause identified by open-source intelligence, 2020–2022

Inclusion criteria: all criteria must be met	Exclusion criteria: any 1 criterion must be met
Article describes a syndromic outbreak in humans or animals.	Article does not describe a syndromic outbreak, and case numbers not mentioned.
Title contains >1 search term, and article published Dec 31, 2019–Jan 1, 2023.	Infectious or noninfectious cause of syndrome identified in article.
Article includes case numbers and location.	Article not accessible.
Cause of syndrome is unknown, and article describes an outbreak in <3 countries.	Article outlines routine monthly, quarterly, or annual syndromic surveillance.
	Article outlines multicountry (>3) syndromic outbreaks.

### Syndromic Outbreak Cause Identification

To identify whether a cause had been subsequently identified, we followed outbreaks of unknown etiology for 3 months after the initial event date. Doing so involved conducting independent web searches and reviewing data within the EPIWATCH database, mapping the syndromic outbreak to subsequent laboratory-confirmed diseases identified within the same location. We examined selected case studies in more detail, focusing on the timeliness of EPIWATCH data compared with official sources and laboratory confirmation of the outbreak.

### Analyses

We conducted data cleaning and performed statistical analyses by using Stata/BE 17.0 (StataCorp LLC, https://www.stata.com). To assess the number of unique outbreaks described by the reports, we considered different articles to be describing the same event if they discussed a similar number of cases of the same syndrome/symptoms in the same location <30 days apart.

To identify the most frequent syndromic outbreaks for humans and animals reported globally and their locations, we calculated the frequency of reported syndromes and locations of outbreaks. We measured the association of outbreak diagnosis by income status, using a χ^2^ test for high-income economies (HIEs) or low-middle–income economies/upper-middle–income economies (LMIEs/UMIEs). As defined by the World Bank, an HIE has a gross national income (GNI) per capita of >$14,005 (in US dollars), a UMIE has a GNI per capita of $4,516–$14,005, and an LMIE has a GNI per capita of $1,145–$4,515 (*12*). We used ArcGIS Pro v.3.1 (https://www.esri.com) to map the geographic distribution of global reports for animal and human outbreaks.

## Results

For our final analysis, we included 1,404 eligible articles (Figure 1). Of those, 1,257 (89.5%) reported outbreaks among humans and 147 (10.5%) reported outbreaks among and animals. The articles described 310 syndromic outbreaks overall, including 249 (80.3%) affecting humans and 61 (19.7%) affecting animals during the study period. Among outbreaks of unknown etiology, 75,968 human cases of illness and 4,235 deaths were recorded. Of the outbreaks of unknown cause among humans reported from 61 countries and among animals from 21 countries, the largest numbers for both were reported from India, followed by the United States (Table 3).

**Table 3:** Top 5 countries reporting syndromic outbreaks among humans and animals, 2020–2022

Country	No. (%) events
Human	
India	110 (44.2)
United States	13 (5.3)
Bangladesh	13(2.8)
Indonesia	7 (2.4)
Russia	6 (2.4)
Animal	
India	28 (46.7)
United States	5 (8.3)
Kazakhstan	3 (5.0)
Russia	3 (5.0)
United Kingdom	2 (3.3)

Among 249 articles for which the clinical syndrome could be classified, the most commonly reported syndromes in humans were respiratory syndrome (15.3%; n = 38), febrile syndromes (15.3%; n = 38), and acute gastroenteritis (14.5%; n = 36) (Table 4). Among reported clinical signs for 417 outbreaks among humans, the most frequent were fever (21.6%; n = 90), diarrhea (14.9%; n = 62), and vomiting (13.4%; n = 56) (Table 5). For 43% of syndromic outbreaks, the sign/symptom information provided in articles reviewed was inadequate for classifying the syndrome.

**Table 4:** Frequency of reported syndromes during outbreaks of unknown cause identified by open-source intelligence, 2020–2022*

Disease or syndrome	Frequency, no. (%)
Unknown	107 (43.0)
Respiratory syndrome	38 (15.3)
Febrile syndrome	38 (15.3)
Acute gastroenteritis	36 (14.5)
Meningitis	16 (6.4)
Fever with rash	7 (2.8)
Encephalitis syndrome	6 (2.4)
Acute flaccid paralysis	1 (0.4)

*Total 249 reports with data on syndromes.

**Table 5:** Ten most frequent signs/symptoms reported during outbreaks of unknown cause identified by open-source intelligence, 2020–2022*

Symptoms	Frequency, no. (%)
Fever	90 (21.6)
Diarrhea	62 (14.9)
Vomiting	56 (13.4)
Breathing difficulties	41 (9.8)
Headache	22 (5.3)
Cough	16 (3.8)
Nausea	16 (3.8)
Stomachache	13 (3.1)
Peripheral swelling	11 (2.6)
Hemorrhagic fever	10 (2.4)

*Total 417 outbreaks with clinical signs/symptoms reported.

**Figure 2:**
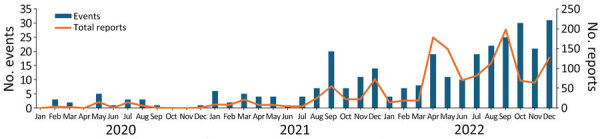
Total reports and events that met study inclusion criteria, by month, for study of global epidemiology of outbreaks of unknown cause identified by open-source intelligence, 2020–2022. Scales for the y-axes differ substantially to underscore patterns but do not permit direct comparisons.

**Figure 3:**
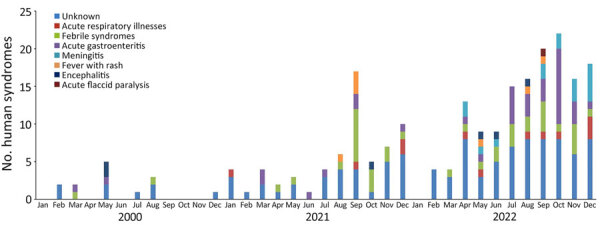
Frequency of human syndromic events, by month, identified in study of global epidemiology of outbreaks of unknown cause identified by open-source intelligence, 2020–2022.

**Figure 4:**
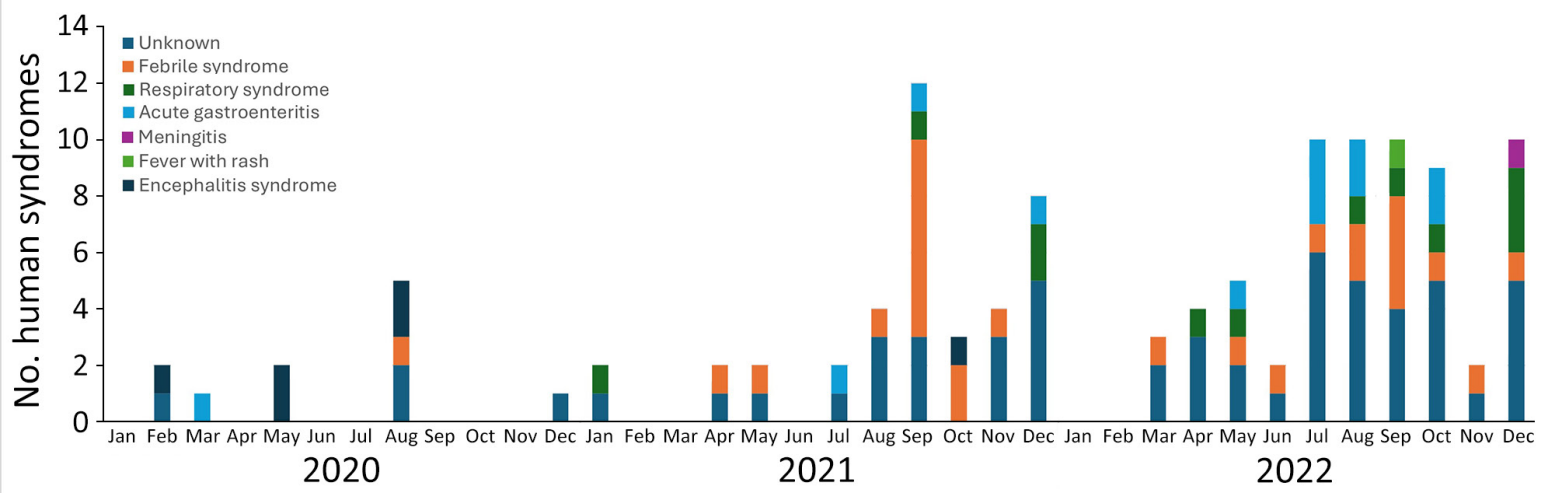
Frequency of human syndromes reported in events in India, by month, identified in study of global epidemiology of outbreaks of unknown cause identified by open-source intelligence, 2020–2022.

Reports of syndromic illnesses increased notably over the study period, from 16 reports in 2020 to 69 reports in 2021 and 171 reports in 2022 (Figures 2, 3). Although outbreaks associated with all syndromes seem to have increased during that period, the increases seemed to be driven primarily by increased gastrointestinal syndromes and syndromes that could not be classified. Respiratory syndromes seem to have been suppressed in 2020, consistent with the known suppression of respiratory infections during 2020 resulting from COVID-19 mitigation efforts. We also noted seasonal patterns of syndromes in particular regions (e.g., febrile syndrome reports in India peaked during July–September, and respiratory syndrome reports peaked in December) (Figure 4).

For only 32 (12.9%) of 249 syndromic outbreaks was a cause subsequently reported for humans, and for 14 (23.0%) of 61 syndromic outbreaks was a cause subsequently reported for animals. The 5 most commonly identified causes of syndromic outbreaks among humans were norovirus infection (15.2%; n = 5), bronchiolitis (6.1%; n = 2), carbon monoxide poisoning (6.1%; n = 2), malaria (6.1%; n = 2), and meningococcal infection (6.1%; n = 2) (Table 6). The proportion of diagnoses was higher in HIEs (40%) than in LMIEs/UMIEs (11%; p<0.001) (Table 7).

**Table 6:** Five most frequent causes of 32 reports with listed cause found during analysis of global epidemiology of outbreaks of unknown cause identified by open-source intelligence, 2020–2022

Disease found during analysis	Frequency, no. (%)
Norovirus infection	5 (15.1)
Bronchiolitis	2 (6.1)
Carbon monoxide poisoning	2 (6.1)
Malaria	2 (6.1)
Meningococcal infection	2 (6.1)

**Table 7:** Proportion of syndromes diagnosed across high and lower-middle and upper-middle–income economies identified during analysis of global epidemiology of outbreaks of unknown cause identified by open-source intelligence, 2020–2022*

Cause	Income, no. (%)		Total
	High	LMIE/UMIE	
Unknown	29 (60)	235 (89)	264 (85)
Known	19 (40)	28 (11)	47 (15
Total	48 (100)	263 (100	311 (100)

*Pearson χ^2^(1) = 26.49; p<0.0001. LMIE, low-middle-income economies; UMIE, upper-middle-income economies.

### Outbreaks in India

The 110 outbreaks of unknown cause in India were identified across different states; several outbreaks affected predominantly children, with no cause found for most. On August 16, 2020, an outbreak of fever and throat swelling was recorded in Bageshwar, Uttarakhand, where 43 children were affected, 6 of whom were admitted to local hospitals (*13*). On April 9, 2021, another outbreak of fever and stomachaches was reported in Uttar Pradesh; 60 children died, and several hundred were hospitalized (*13*). Although dengue fever was suspected in that outbreak, it was not confirmed. On August 24, 2021, in Mathura, Uttar Pradesh, 6 children, 5–15 years of age, died of an unknown illness over a 1-week period (*14*). On June 22, 2022, a large outbreak of fever among 250 persons from Kanakatte, Karnataka, was recorded; signs/symptoms included headaches, blisters, and joint pain (*15*). On July 24, 2022, in Sheopur, Madya Pradesh, 3 persons died and 15 other persons were reportedly ill with gastroenteritis and fever of unknown cause (*16*). On August 31, 2022, in Garhwal village, Uttarakhand, a mysterious disease was reported of which >100 persons fell ill with fever, chest pain, vomiting, and pain in joints of their hands and feet (*17*). The large numbers of cases and fatalities (including among children) and lack of clear causes found for all of those outbreaks highlight the need for syndromic surveillance to guide outbreak investigation and diagnostic testing.

### Pneumonia in Argentina

During August 18–22, 2022, EPIWATCH identified reports of bilateral pneumonia in a cluster of 3 hospitalized patients in San Miguel de Tucumán City, Tucuman Province, Argentina. Early cases were among healthcare workers, resulting in their admission to intensive care units. Six days later, on August 29, 2022, the Argentine Ministry of Health of Tucumán Province notified the World Health Organization (WHO) of a cluster of 6 cases of bilateral pneumonia lacking cause; 1 patient died on August 30, 2022 (*18*). On September 3, 2022, the cause was identified as *Legionella pneumophila* and *Legionella* spp. (*19*), although the cause was only officially reported by WHO on September 5, 2022, prompting an investigation of the source, which identified 22 suspected cases and 6 fatalities. Four of the patients tested positive for *L. pneumophila* and *Legionella* spp*.* and had clinically compatible illnesses (*19*). We note that EPIWATCH identified this syndromic outbreak before it was reported through official sources.

### Hepatitis among Children, Worldwide

On March 31, 2022, severe acute hepatitis of unknown origin in children was reported for the first time among children in Scotland for causes other than common hepatitis A–E virus infection; patients experienced jaundice, vomiting, gastrointestinal symptoms, and fever (*20*). On April 6, 2022, EPIWATCH identified reports of 11 children, 1–5 years of age, who were receiving treatment for a hepatitis-like infection with jaundice as the primary syndrome (*21*). On April 15, 2022, WHO released its first related report, stating that 10 cases across the United Kingdom and Europe were detected on April 5, 2022 (*22*). Two retrospective studies that analyzed surveillance data in Japan and Israel showed possible syndrome onset as early as September and October 2021 (*23*,*24*). By August 2022, a total of 35 countries reported 1,115 cases of acute hepatitis of unknown origin that fulfilled the WHO definition (*25*). Among those patients, 47 (4%) of children required liver transplants because of organ failure associated with infection and 22 (2%) deaths were reported (*25*). Only 479 case reports contained information for age and sex; 78% of patients were <6 years of age, and 48% were boys and 52% girls (*25*). Investigations to determine the potential cause of acute hepatitis of unknown origin around the world are ongoing. However, limited evidence suggested a postinflammatory syndrome with potential links to SARS-CoV-2 infection (*25*), with stronger evidence for SARS-CoV-2 being associated with this syndrome. 

### Pneumonia in Kazakhstan

On July 9, 2020, an unusual increase in cases of pneumonia of unknown cause in Kazakhstan was reported (*26*). The increase began in January 2020 at the early stages of the COVID-19 pandemic. Although 264 deaths from COVID-19 had been reported in the country until July 2020, Kazakhstan reported 1,772 deaths in the first 6 months of 2020 from pneumonia of unknown cause; 628 were reported in June alone (*27*). Reports stated that 300 persons were being hospitalized daily and up to 600 new cases of pneumonia were reported daily, compared with an average of 80 cases per day before the outbreak of COVID-19 (*27*). In March 2020, Kazakhstan implemented a short, sharp lockdown because of the COVID-19 outbreak, and at the end of May 2020, quarantine measures were lifted (*28*), supporting the hypothesis that the increased pneumonia cases were probably directly associated with a resurgence of COVID-19 cases.

## Discussion

During 2020–2022, outbreaks of unknown illnesses affecting human and animal populations occurred regularly; for most outbreaks, a cause was never identified. However, outbreaks with unidentified causes will not be formally reported because national notifiable disease systems are for identified diseases only. Most outbreak patients exhibited sign/symptoms consistent with infectious causes; however, the cause of some outbreaks may be noninfectious (e.g., chemical exposure). Open-source syndromic surveillance provides a unique means for understanding the global epidemiology of outbreaks of unknown etiology and probably represents the only available public record of those illnesses.

We describe examples in which EPIWATCH OSINT detected an early signal of an unknown respiratory outbreak with fatalities before an official diagnosis was made (e.g., pneumonia subsequently diagnosed as legionellosis in Argentina). We also describe instances in which syndromic surveillance can provide possible indicators of a surge of known disease with pandemic potential in areas where diagnostic capability is limited (e.g., pneumonia surge, thought to be caused by COVID-19, in Kazakhstan). In India, no cause has been identified for recurrent, large outbreaks of encephalitis in certain states, such as Bihar (*29*). Those findings highlight the vital role of syndromic illness surveillance and OSINT to bridge the gap between formal surveillance and provide early warnings of syndromic outbreaks before diagnosis. Early warnings can enhance rapid response (e.g., quarantine) while laboratory diagnosis is in progress.

Approximately 80% of outbreaks assessed in our final analysis did not have a cause subsequently identified or reported, possibly because of lack of formal diagnosis, media reporting, or censorship (*30*). Outbreaks for which a cause was eventually identified were more frequent in HIEs with well-developed surveillance systems (Table 7). That finding highlights the vital role of syndromic surveillance, particularly in LMIEs, where disease-based surveillance may be less robust. Even in HIEs, the cause of most outbreaks of unknown cause was not identified or reported. OSINT is useful for providing early intelligence to public health officials for targeted outbreak investigations independent of formal surveillance.

Over the 3-year study period, we observed increased reports of unknown syndromic illnesses, which may be attributable to the upscaling of the EPIWATCH system, increased reporting of syndromes after the COVID-19 pandemic, or real increases in illness. COVID-19 mitigation strategies contributed to decreased incidence of respiratory and other illnesses, which our finding may reflect (*31*). However, in recent years, respiratory infections have begun to increase, including respiratory syncytial virus, influenza virus, and SARS-CoV-2 (known as the tripledemic) infection.

**Figure 5:**
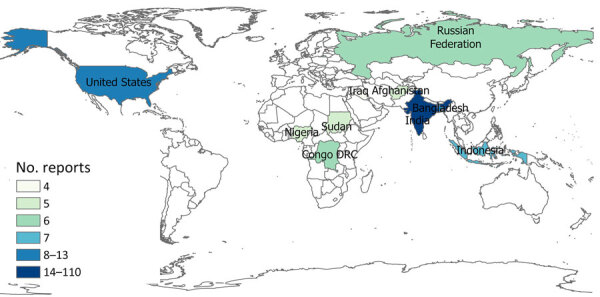
Geographic distribution of top 10 countries reporting unknown illnesses in humans, identified in study of global epidemiology of outbreaks of unknown cause identified by open-source intelligence, 2020–2022.

**Figure 6:**
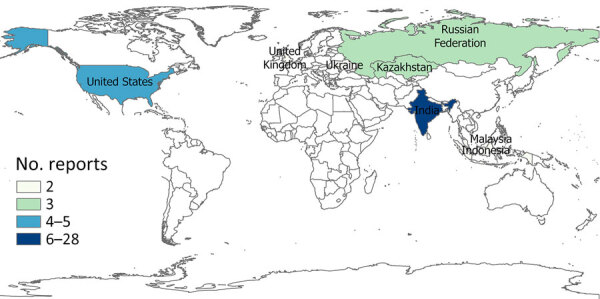
Geographic distribution of top 8 countries reporting unknown illnesses in animals, identified in study of global epidemiology of outbreaks of unknown cause identified by open-source intelligence, 2020–2022.

The geographic distribution of unknown illnesses affecting humans and animals indicates that outbreaks were reported most frequently from India and the United States (Figures 5, 6). That finding may be attributable to higher coverage by print and electronic media, inclusion of multiple Indian languages in EPIWATCH, and increased reporting of infectious diseases in some locations (*32*), which could be contributing to increased signals in India. However, our results are conservative because of the way events have been inferred from articles and the use of OSINT. Our estimates represent the lower end of reported outbreaks of unknown cause.

The strengths of our study include use of OSINT to detect early epidemic signals, especially in countries with poor surveillance capacity (*33*). Use of OSINT overcomes issues associated with formal surveillance and diagnostic dependence, providing a means for syndromic surveillance. The strength of EPIWATCH lies in its capacity to search in 46 languages, incorporating Natural Language Processing, which seeks to offset, to some extent, the Anglo-centric bias inherent in OSINT and search algorithms (e.g., Google). To our knowledge, EPIWATCH is the most comprehensive publicly available OSINT platform to date (*33*). As an example of an early warning, in November 2019 before COVID-19 was identified, we previously showed a signal of unknown pneumonia in Wuhan, China, as well as evidence of redacted news reports with “SARS” in the title (*8*). EPIWATCH is not directly comparable with ProMED Mail, which is qualitative and relies on reports of outbreaks from clinicians in the field, rather than OSINT (*34*). Both are valuable sources of early warning.

The limitations of our study include the fact that OSINT, as used in our study, ultimately depends on reporting patterns and search algorithms, which may change over time, may prioritize information in biased ways, and may not capture every early signal. Therefore, changes over time and differences in geographic reporting may reflect differences in media practice and search algorithms rather than actual disease burden. OSINT relies heavily on publication of correct information, which requires further analysis to determine if outbreaks are accurately reported. Another limitation is the short duration of follow-up with no data from the prepandemic period in this study, making the effects of the pandemic and mitigations during the pandemic difficult to assess. Another limitation of our results is that the true number of events within countries has not been corrected for population size.

Although similar work using the EPIWATCH system has been previously conducted (*4*), the search terms and algorithms used were not comparable to those that we used. Syndromic surveillance (focusing on diseases of unknown cause) is likely to capture different diseases in different regions (*35*). Differences observed with syndromic surveillance may reflect resources available for ascertainment rather than disease burden. Data are frequently reported from a country level, meaning granular analysis is difficult because of a lack of confirmed province, state, or city. Geopolitical factors may also influence the number of reports. For example, war or other events may increase surrounding health issues and subsequent regional reporting (*36*). The purpose of OSINT in that context should be seen not as a tool to provide diagnostics but rather as an adjunct to formal surveillance and an early warning system to alert authorities to potential threats and guide outbreak responses.

Our study using EPIWATCH provides a global surveillance data resource enabling public health professionals to assess hot spots of unknown disease outbreaks and the presence of potential new unknown illnesses as early warning signals to be used to enhance surveillance and reporting capacities. EPIWATCH provides a framework for open-source syndromic surveillance for public health agencies, especially in low-income and under-resourced settings where formal surveillance systems are not adequately linked. The EPIWATCH system aims to identify outbreak signals before government authorities are aware of them. As such, it should be an adjunct to formal surveillance rather than a replacement, with the understanding that the data are not validated. Where national health organizations fail to disclose outbreaks of concern internationally, web-based syndromic surveillance can act as the first line of defense for surrounding countries to encourage the initiation of outbreak response and investigation to prevent the next pandemic.

In conclusion, using OSINT for syndromic surveillance from the EPIWATCH system, we found early signals of human and animal unknown illnesses across 310 outbreaks. The cause was not identified for most outbreaks, especially in LMIEs. Our work highlights the value of OSINT-based digital surveillance systems for identifying syndromic outbreaks and guiding rapid outbreak response.
